# The Protective Role of Dietary Polyphenols in Urolithiasis: Insights into Antioxidant Effects and Mechanisms of Action

**DOI:** 10.3390/nu15173753

**Published:** 2023-08-28

**Authors:** Sen-Yuan Hong, Bao-Long Qin

**Affiliations:** Department of Urology, Tongji Hospital, Tongji Medical College, Huazhong University of Science and Technology, Wuhan 430030, China

**Keywords:** urolithiasis, oxidative stress, dietary polyphenols, antioxidants

## Abstract

Urolithiasis is a common urological disease with increasing prevalence and high recurrence rates around the world. Numerous studies have indicated reactive oxygen species (ROS) and oxidative stress (OS) were crucial pathogenic factors in stone formation. Dietary polyphenols are a large group of natural antioxidant compounds widely distributed in plant-based foods and beverages. Their diverse health benefits have attracted growing scientific attention in recent decades. Many literatures have reported the effectiveness of dietary polyphenols against stone formation. The antiurolithiatic mechanisms of polyphenols have been explained by their antioxidant potential to scavenge free radicals and ROS, modulate the expression and the activity of endogenous antioxidant and prooxidant enzymes, regulate signaling pathways associated with OS, and maintain cell morphology and function. In this review, we first describe OS and its pathogenic effects in urolithiasis and summarize the classification and sources of dietary polyphenols. Then, we focus on the current evidence defining their antioxidant potential against stone formation and put forward challenges and future perspectives of dietary polyphenols. To conclude, dietary polyphenols offer potential applications in the treatment and prevention of urolithiasis.

## 1. Introduction

Urolithiasis is a prevalent urological disease characterized by the formation of solid mineral and salt crystals (commonly known as calculi or stones) within the urinary tract [1]. The prevalence of urolithiasis is on the rise worldwide, which varies across different regions and populations [2]. Around 10.6% of men and 7.1% of women in the United States experience kidney stones in their lifetime [3]. Additionally, 67% of patients will experience a stone recurrence within 5 years after the first episode [4]. Recurrent episodes of stones could cause severe renal damage and gradually develop into chronic renal failure, which constitutes a notable health and economic burden on individuals and society [5]. Recent years have witnessed a rapid development of minimally invasive surgery, greatly improving the stone removal efficiency [6]. However, the development of pharmaceutical drugs to treat and prevent urolithiasis is almost stagnant, as the pathogenesis of stone formation is not completely clear.

Most urinary calculi are calcium stones, accounting for 80% of all stones, and calcium stones consist of pure calcium oxalate (CaOx), calcium phosphate (CaP), and a mixture of both [7]. Uric acid, struvite, and cystine stones accounted for the remainder [7]. In the physicochemical theory, stone formation involves a sequence of events, including urine supersaturation, crystal nucleation, growth, aggregation, and retention [8]. Hypercalciuria, hyperoxaluria, and hypocitraturia are major risk factors for stone formation, which could trigger urinary supersaturation and induce the formation of CaOx or CaP crystals in the renal tubular lumen [9,10]. CaOx crystals can attach to renal tubular epithelial cells and be internalized into cells by endocytosis [11]. This crystal-cell interaction has been recognized as a critical step in stone formation [12]. In addition to this theory, Randall’s plaques (RPs) theory is another accepted theory. RPs are defined as the presence of CaP crystal deposits in the renal interstitium, which gradually expand and extend until they reach the renal papillary surface, becoming exposed to pelvic urine. Subsequently, CaOx crystals adhere to these exposed sites and progress into CaOx stones [13,14]. Numerous studies have reported the pivotal involvement of reactive oxygen species (ROS) and oxidative stress (OS) in stone formation, both in these two theories [15].

Dietary polyphenols are a large group of naturally occurring compounds found in various plant-based foods and beverages [16]. Dietary polyphenols can be classified into several classes, including flavonoids, phenolic acids, stilbenes, lignans, and others [17]. Foods rich in dietary polyphenols include fruits, vegetables, legumes, nuts, and beverages, such as green tea, coffee, and red wine [18]. Dietary polyphenols are a type of phytochemical known for their potential health benefits, such as antioxidant activity, anti-inflammatory activity, anti-cancer, and anti-microbial activity, which have been the subject of extensive research in the fields of nutrition and medicine [19]. The presence of aromatic structural features and multiple hydroxyl groups, coupled with a highly conjugated structure, enables dietary polyphenols to efficiently scavenge free radicals and ROS, thereby mitigating OS and preserving redox homeostasis [20]. Much evidence indicates that dietary polyphenols offer protection against urolithiasis. In this review, we start by introducing OS and its role in urolithiasis and presenting the classification, sources, and functions of dietary polyphenols. Then, the current research progress on the protective role of dietary polyphenols in urolithiasis is reviewed and summarized. On this basis, challenges and future perspectives of dietary polyphenols will be put forward.

## 2. The Role of Oxidative Stress in Urolithiasis

### 2.1. Sources of Reactive Oxygen Species in Kidney

The term ROS refers to free radicals (atoms and molecules with unpaired electrons) as well as their metabolites. Major ROS include superoxide anion radical (O^2−●^), nitric oxide radical (NO^●^), hydroxyl radical (OH^●^), and hydrogen peroxide (H_2_O_2_) [21]. ROS are produced through tightly regulated enzymes under physiological conditions and function as mediators in diverse regulatory processes and signaling pathways. They normally occur at steady-state levels and are then removed by antioxidants and scavengers [22]. There are several antioxidant enzymes that remove free radicals, such as superoxide dismutase (SOD), catalase (CAT), and reduced glutathione (GSH) [23]. However, under pathologic situations, overproduction of ROS and a decrease in endogenous antioxidant capacity will lead to OS, which may further induce inflammation and injury [24,25].

The major source of ROS in the kidney is NADPH oxidase [26,27]. NADPH oxidases can be classified into seven isoforms based on their catalytic subunit: NOX1–5, DUOX1, and DUOX2 [28]. NOX2 and NOX4 are the two main isoforms expressed in the kidney, and NOX4 is most abundant in the kidneys in various kinds of cells [27,29]. NOX2 shares many structural similarities with most NOX isoforms, which comprises six subunits, including two transmembrane units, gp91phox and p22phox, and four cytosolic units, p47phox, p67phox, p40phox, and the small GTPase rac1 or rac2 [30]. NOX2 can be activated when the cytosolic units translocate to the membrane and assemble with the cytochrome, while NOX4 appears constitutively active and does not require cytosolic subunits [31].

Mitochondria are also involved in the generation of ROS, an important source of O^2−●^ and H_2_O_2_ [32]. The electron transport chain (ETC) within the inner mitochondrial membrane is responsible for the transfer of the majority of electrons, resulting in ATP production. However, a small proportion of electrons may prematurely escape the ETC and interact with molecular oxygen (O_2_) within the mitochondria, generating O^2−●^. Subsequently, O^2−●^ can undergo additional reactions to generate other reactive oxygen species (ROS), such as H_2_O_2_ and OH• [33]. The excessive accumulation of ROS within mitochondria can trigger the opening of mitochondrial membrane channels (inner membrane anion channels and mitochondrial permeability transition pores), which in turn promotes the overproduction of ROS and mitochondrial damage, establishing a detrimental cycle [34].

### 2.2. Clinical and Experimental Studies of Oxidative Stress in Urolithiasis

Several clinical studies on the urine analysis of stone formers indicated that OS and OS-induced renal injury and inflammation might be implicated in urolithiasis. CaOx stone formers exhibited increased levels of tubular injury indicators, like γ-glutamyl transpeptidase, angiotensin 1 converting enzyme, β-galactosidase, and NAG, in their urine [35]. Urinary 8-hydroxydeoxyguanosine, a marker of DNA oxidative damage, was found to be higher in stone formers and positively correlated with tubular damage [36]. Schwille et al. reported an association between stone formation and reduced antioxidant levels in recurrent calcium stone formers [37]. Holoch et al. found that participants with a self-reported history of kidney stones showed decreased levels of antioxidants, like α-carotene, β-carotene, and β-cryptoxanthin [38]. Previously, it was assumed that RPs are formed without causing renal injury and inflammation [39]. However, some researchers have observed injured and necrotic tubules around the interstitial deposits of CaP crystals [40,41].

Many animal studies have been conducted to explore the pathogenesis of stone formation, which supported the involvement of OS in urolithiasis. CaOx stone models are established by inducing hyperoxaluria via the administration of Ox or its precursors, such as glyoxylate, ethylene glycol, and hydroxyl-L-proline [42]. Hyperoxaluria and CaOx crystal deposition can cause several morphological and pathophysiological changes in the kidneys and urine of animal models, serving as indicators of inflammatory responses in renal cells exposed to elevated levels of Ox and CaOx crystals [42,43]. Rat models exhibited increased expression of kidney injury molecule and NF-κB in the renal tubules around deposits of CaOx crystals [43]. Rat models also showed increased levels of lipid peroxides in both the renal tissue and urine [44]. Additionally, inflammatory immune cells, including leukocytes, monocytes, macrophages, and multinucleated giant cells, were observed around CaOx crystal deposition [45,46].

In vitro studies also offer insight into the role of OS in stone formation. Renal epithelial cells can generate excessive ROS derived from NADPH oxidases and mitochondria after exposure to Ox or CaOx crystals. HK-2 cells exposed to Ox and CaOx crystals showed increased expression of membrane-bound p22phox and cytosolic p47phox, along with higher NADPH oxidase activity and superoxide production and LDH release [47]. Thamilselvan et al. confirmed that Ox activated NADPH oxidase through Rac-1 and PKC-α and -δ [48,49]. Byer et al. found that mitochondria are a major source of superoxide production and glutathione depletion in LLC-PK1 and MDCK cells under stimulation of CaOx crystals [50]. Isolated mitochondria induced the accumulation of ROS, lipid peroxides, and oxidized thiol proteins in response to Ox exposure [50]. The imbalance of cellular oxidants and antioxidants leads to OS and further promotes the expression of specific transcriptional activators, crystallization modulators, and inflammatory molecules, such as NF-κB, AP-1, OPN, MCP-1, CD-44, and others [51,52,53]. For example, OPN can accelerate crystal adhesion and deposition and induce macrophage infiltration around deposition [54]; CD44 can interact with OPN to promote crystal retention within the kidneys [55].

**Figure 1:**
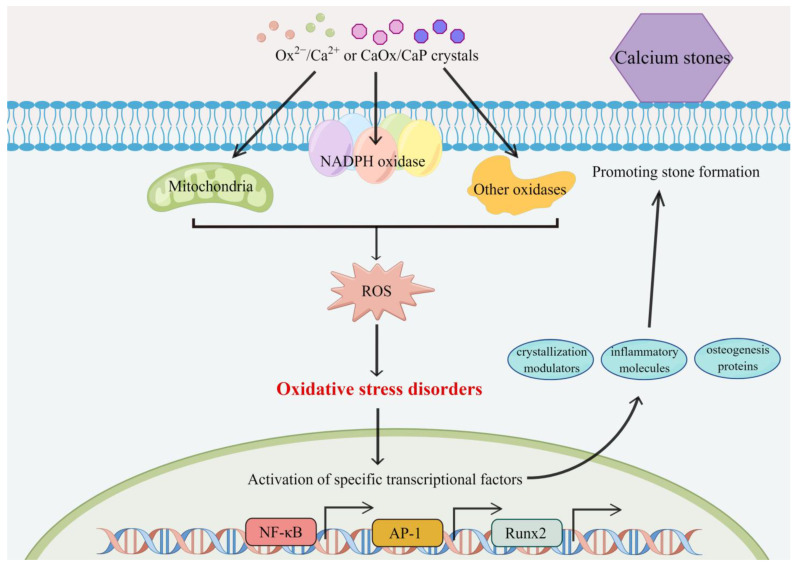
Proposed scheme for the involvement of ROS and OS and their pathogenic roles in urolithiasis.

Notably, renal epithelial cells under OS may transform into osteogenic phenotypes. Khan et al. found that osteogenesis-related gene expression (Runx2, Osterix, BMP2, OPN, OCN, collagen) was increased in the kidneys of rat models [56]. Similarly, Okada et al. observed the upregulation of osteogenesis-related gene expression in a mouse model [57]. Exposure to calcium also leads to osteogenic changes in the renal epithelial cells and might release calcified vesicles into the renal interstitium, facilitating the formation of RPs [14]. In addition, persistent OS might result in severe cell damage, even death. On the one hand, dead cells and membrane vesicles produced from cell degradation are good promotors of crystal nucleation [58]. On the other hand, cell death also leads to the formation of new cells to repopulate the epithelium, acting as the suitable surface for crystal attachment and retention [53]. Figure 1 shows the involvement of ROS and OS and their pathogenic roles in urolithiasis.

### 2.3. Antioxidants for Treatment

The above evidence indicates that OS plays a pivotal pathogenic role in the formation and development of urinary calculi. Thus, antioxidants have been investigated as potential alternatives for the prevention and treatment of urolithiasis. Antioxidants have already exhibited protection against CaOx monohydrate (COM)-induced OS. The treatment with vitamin E in rat models led to a significant improvement in renal antioxidant enzyme levels, a reduction in peroxidative tissue injury, and elimination of CaOx crystal deposition in the kidneys [59]. *N*-acetylcysteine alleviated crystal deposition and improved renal function by maintaining the redox balance [60]. Jeong et al. found that the activities of antioxidant enzymes (SOD, CAT, GSH) were recovered with the supplementation of green tea, thereby reducing crystal deposition [61]. It is known that green tea contains a rich array of dietary polyphenols, which have strong antioxidant capacities.

## 3. Dietary Polyphenols and Their Biological Significance

### 3.1. Classification and Sources of Dietary Polyphenols

Polyphenols are secondary metabolites characterized by one or more aromatic rings with one or more hydroxyl groups, which are widely distributed in roots, stems, leaves, flowers, fruits, and seeds of plants [62]. Dietary polyphenols are classified into four major groups based on the number of aromatic rings they contain and the structural elements that connect these rings, including flavonoids, phenolic acids, stilbenes, and lignans [63]. Flavonoids comprise a series of phenolic compounds with low molecular weight polyphenolic structures. Flavonoids have a fundamental C6-C3-C6 structural backbone consisting of two benzene rings connected by a third heterocyclic oxygen-containing pyrene ring [64]. Based on variations in their chemical structures, flavonoids can be further divided into six subgroups, including flavones, flavanols, flavanones, flavonols, isoflavones, and anthocyanins [65]. Phenolic acids are characterized by the presence of a carboxylic acid group linked to the phenolic ring, and they are divided into two subgroups, namely, hydroxybenzoic acids (C6-C1 backbone) and hydroxycinnamic acids (C6-C3 backbone) [66]. Stilbenes are identified by a 1,2-diphenylethylene nucleus consisting of two phenolic rings connected by a methylene bridge (C6-C2-C6) [67]. The most well-known stilbene is resveratrol, which has received significant attention for its potential health benefits [68]. Lignans are generated from shikimic acid via the phenylpropanoid pathway through the oxidative dimerization of two phenylpropanoid C6-C3 units [69]. Other groups of polyphenols are curcuminoids, such as curcumin, and tannins, such as condensed tannins and hydrolyzable tannins [70,71]. Overall, flavonoids and phenolic acids are the most abundant polyphenolic compounds in food [72]. Table 1 exhibits different groups and subgroups of dietary polyphenols and their sources.

### 3.2. Antioxidant Mechanisms of Dietary Polyphenols

**Figure 2:**
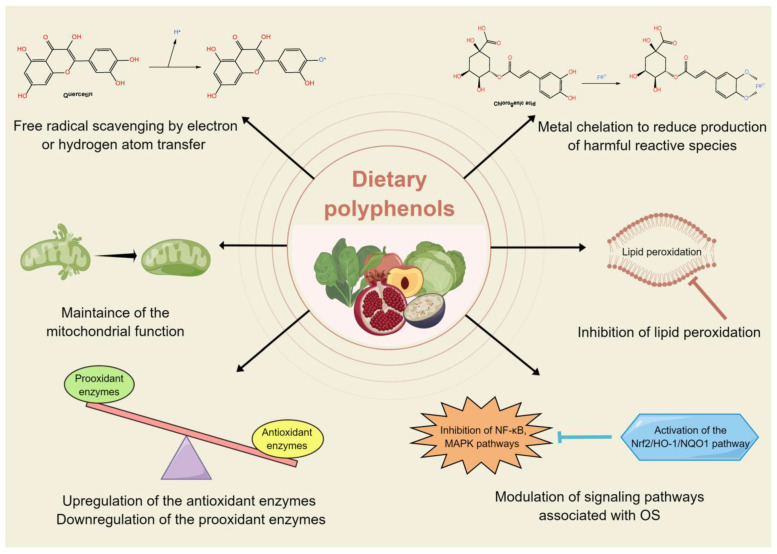
Mechanisms involved in the antioxidant potential of dietary polyphenols.

Dietary polyphenols exert a wide range of bioactivity, such as antioxidant activity, anti-inflammatory effects, anti-cancer properties, cardiovascular and neuroprotective benefits, and others, holding health-protecting effects for human wellness [17,73]. The bioactivity of polyphenols is contingent on their activity levels and the extent of their distribution, metabolism, absorption, and elimination from the body [74]. Numerous studies have demonstrated the great antioxidant potential of dietary polyphenols. First, polyphenols can directly interact with and neutralize free radicals. They donate electrons or hydrogen atoms to these radicals, stabilizing them and converting them into less harmful species [75]. Second, some polyphenols have metal-chelating properties. By chelating transition metals like iron and copper, which are catalysts for the generation of highly reactive free radicals, polyphenols can prevent or reduce the production of harmful reactive species [76]. Third, certain polyphenols can stimulate the expression and activity of endogenous antioxidant enzymes, such as SOD, CAT, and GSH, and inhibit the activity of enzymes that produce free radicals or promote OS within cells, such as xanthine oxidase, myeloperoxidase, NADPH oxidase [77]. Fourth, polyphenols can interact with signaling pathways related to OS and inflammation. By modulating these pathways, they may reduce the production of ROS and inflammatory mediators, contributing to their overall antioxidant and anti-inflammatory effects [63]. For example, the Nrf2 pathway is a cellular defense mechanism against OS. Polyphenols have the ability to activate the Nrf2 pathway by facilitating the translocation of Nrf2 protein into the nucleus, thereby inducing the expression of downstream antioxidant proteins, including heme oxygenase-1 (HO-1) and NAD(P)H quinone dehydrogenase 1 (NQO1), which effectively mitigate cellular OS disorders [78]. Fifth, polyphenols can maintain mitochondrial function and form by maintaining mitochondrial ATP output and calcium homeostasis to prevent further aggravation of OS [79]. In addition, polyphenols can inhibit lipid peroxidation, a process in which free radicals attack and damage lipids in cell membranes. By preventing lipid peroxidation, polyphenols help maintain cell membrane integrity and function [80]. Figure 2 outlines the mechanisms involved in the antioxidant potential of dietary polyphenols.

## 4. Antioxidant Potential of Dietary Polyphenols in Urolithiasis: In Vitro and In Vivo Studies

### 4.1. Flavonoids Compounds

Flavones: Azimi et al. found apigenin exhibited great antioxidant activity and reduced crystal deposition in urolithiatic rats via inhibition of the TGF-β pathway [81]. Vitexin, namely apigenin 8-C-glucoside, was reported to inhibit pyroptosis, the epithelial–mesenchymal transition of renal tubular epithelial cells, and macrophage infiltration to alleviate crystal deposition and renal OS injury [82].

**Figure 3:**
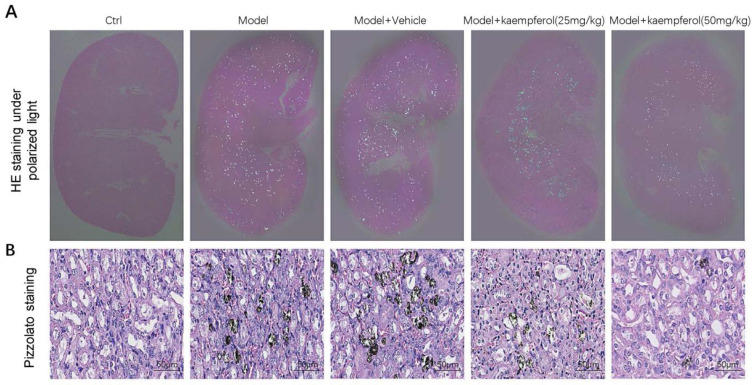
Representative pictures on the protective effects of kaempferol against urolithiasis. CaOx crystal deposition was verified by polarized light microscopy (**A**) and von Pizzolato staining (**B**).

Flavonols: Park et al. first reported the antioxidant property of quercetin in urolithiasis via inhibition of lipid peroxidation and activation of SOD and CAT activities [83]. Guzel et al. found quercetin might inhibit the p38-MAPK pathway to suppress OS [84]. Quercetin also can maintain a tighter epithelial barrier to inhibit the reabsorption of sodium, calcium, and water and further prevent stone formation [85]. Hyperoside, also called quercetin 3-O-galactoside, can improve the OS injury of HK-2 cells treated with oxalate via activation of the Nrf2/HO-1/NQO1 pathway [86]. Zhu et al. combined quercetin and hyperoside to treat urolithiatic rats, showing a great inhibitory effect on crystal deposition [87]. Yuan et al. found androgen receptor (AR) can directly bind to the promoter of NOX2 to upregulate its expression at the transcriptional level, and kaempferol alleviated CaOx crystal-induced OS and deposition via suppression of the AR/NOX2 pathway [88]. Figure 3 showed there were much fewer renal CaOx crystals in the kaempferol treatment group than in the model group. In addition, significantly decreased CaOx crystal deposition along with increased kaempferol concentration was observed [88].

Flavanols: Zhai et al. conducted a study to investigate the antioxidant properties of catechin in urolithiasis. Their findings revealed that catechin effectively prevented alterations in mitochondrial membrane potential, leading to improved mitochondrial function. Additionally, catechin demonstrated the ability to reduce lipid peroxidation and inhibit the expression of apoptosis-related genes in NRK-52E cells exposed to COM [89]. In vivo studies also showed that catechin treatment repaired antioxidant defenses and prevented crystal deposition [89]. Catechin also alleviated melamine-induced crystal deposition via inhibition of OS, apoptosis, p-p38, and OPN expression in rats [90]. Jeong et al. found that epigallocatechin gallate (EGCG), a main compound of green tea, reduces ROS production in NRK-52E cells exposed to oxalate [61]. Grases et al. showed that catechin and epicatechin treatment were reported to reduce calcium concentration in kidney tissue of rat models [91]. Thongboonkerd et al. observed that EGCG reduced the cell-surface expression of alpha-enolase, a protein with a high affinity for CaOx crystals. Consequently, this reduction in alpha-enolase expression resulted in a decrease in crystal binding onto renal tubular cells and subsequent crystal deposition [92]. They also observed that EGCG protects against microvillar injury in COM-treated renal tubular cells by suppressing the expression of oxidized proteins [93]. Theaflavin, a prominent flavanol derived from black tea, was observed to interact with miR-128-3p, impeding its direct binding to the 3’-UTR of the target gene SIRT1. This interaction ultimately leads to the promotion of SIRT1 expression, which possesses antioxidant and anti-inflammatory properties in urolithiasis [94].

Isoflavones: Puerarin, the most abundant isoflavone from the root of Pueraria lobata, was reported to activate the SIRT1/AKT/p38 pathway to inhibit COM-induced OS and autophagy, and treatment with Puerarin significantly attenuates crystal deposition [95].

### 4.2. Non-Flavonoids Compounds

Gallic acid showed great antioxidant properties in urolithiasis via activation of the Nrf2/HO-1 pathway in HK-2 cells treated with COM and further suppressed crystal deposition in rat models [96]. Chlorogenic acid was reported to suppress the NF-κB/Runx2/AP-1/Osterix pathway to alleviate OS injury in rat models [97]. Treatment with caffeic acid could downregulate the expression of OPN and upregulate the expression of prothrombin fragment 1, Tamm–Horsfall glycoprotein, and bikunin to protect against urolithiasis [98]. Several phenolic acid derivatives showed potent inhibitory activity against xanthine oxidase [99]. Resveratrol is a polyphenolic stilbene with antioxidant activity. Hong et al. found that resveratrol suppressed the expression of NADPH oxidase subunits (p22phox and p47phox), MCP-1, OPN, TGF-1, TGFR-I/II, and hyaluronan in oxalate-treated human primary renal epithelial cells to prevent stone formation [100]. Oksay et al. also observed the antioxidant effect of resveratrol in rat models, and they assumed that the p38-MAPK and NF-κB pathways might be involved in the process [101]. Wu et al. found that resveratrol promoted the expression of transcription factor EB (TFEB) in NRK-52E cells treated with oxalate, and TFEB further activated autophagy to inhibit OS and crystal deposition [102]. Ghodasara first reported the antiurolithiatic effect of curcumin, a polyphenolic yellow substance isolated from Curcuma longa, possibly by reducing the urinary concentration of stone-forming constituents [103]. Li et al. found that curcumin activated the Nrf2/HO-1/NQO1 pathway to suppress OS and crystal deposition in mice models [104]. Gallotannin, a type of hydrolyzable tannin found in vegetable diets that possess gallic acid as the basic unit of the polyester, prevents stone formation via inhibition of crystallization modulators expression (MCP-1 and OPN) and its antioxidant activity [105]. Table 2 summarizes the main findings of the antioxidant potential of polyphenol compounds on urolithiasis.

### 4.3. Plant Sources

Researchers also explore the antioxidant potential of polyphenol-rich plants in urolithiasis. Although green tea is an oxalate-rich natural agent, it is also rich in polyphenol compounds, such as flavonoids, phenolic acids, and tannins, which provide clear benefits to human health, like anti-atherosclerotic and anti-tumor effects [106,107]. Itoh et al. found that green tea inhibited the expression of OPN, inhibited renal epithelial cell apoptosis, increased SOD activity, and reduced crystal deposition in rat models [108]. In a study conducted by Li et al., urolithiatic rats were administered green tea, and their kidney’s CaOx crystals were extracted. The researchers discovered that the CaOx crystals from the green-tea-treated model groups primarily consisted of calcium oxalate dihydrate (COD) and exhibited significantly smaller sizes compared to the non-treatment model groups [109]. Since COD has lower cell adhesion ability, crystal depositions were decreased in green-tea-treated model groups. In vitro studies showed that green tea regulates the SRB1 and Nrf2/HO1/NQO1 pathways to inhibit OS [109]. Raspberry is a kind of fruit rich in ellagitannins and anthocyanins. Treatment with raspberry reduced malondialdehyde (MDA, a maker of lipid peroxidation) and protein carbonyl (products of protein oxidation) generation with decreased levels of urinary calcium and phosphorus in mice models [110]. The extracts and juice of pomegranate, a rich source of polyphenolic components, were reported to effectively suppress the production of reactive oxygen species (ROS) and inducible nitric oxide synthase (iNOS), as well as inhibit the NF-κB and p38-MAPK pathways. These actions contribute to the alleviation of renal OS and the deposition of crystals [111,112].

Various medicinal plants and herbs also play a protective role in urolithiasis. Zhang et al. found *Glechomae Herba* exerted antioxidant function to protect against stone formation in urolithiatic rats and further identified four active antiurolithiatic compounds in *Glechomae Herba*, including chlorogenic acid, rosmarinic acid, luteolin, apigenin [113]. The molecular docking results suggested that the above compounds could interact with CASR to inhibit OS [113]. Li et al. also screened 120 active compounds in *Glechomae Herba*, including 10 chlorogenic acids, 10 gallic acids, and 77 flavonoids [114]. The antiurolithiatic properties of lyophilized juice from Viburnum opulus have been attributed to its ability to inhibit oxalate levels and the production of free radicals. Notably, chlorogenic acid has been identified as the primary compound in the juice [115]. *Orthosiphon stamineus Benth*, a traditional medicinal herb mainly containing flavonoids and caffeic acid derivatives, can suppress stone formation by improving OS and inflammation injury via glycerophospholipid metabolism [116]. Zhou et al. extracted the total flavonoid content from leaves of *Desmodium styracifolium* and identified that the major active compounds were vicenin 1, vicenin 2, isoschaftoside, and isovitexin [117]. Treatment with extracts significantly promoted CAT and GSH activities and reduced MDA content in the kidney of urolithiatic rats; additionally, a decrease in MCP-1, OPN, and TGF-β expression was also observed [117].

## 5. Antioxidant Potential of Dietary Polyphenols in Urolithiasis: Clinical Investigation

There have been very few clinical studies exploring the protective effects of polyphenols in urolithiasis. Some retrospective studies reported that green tea consumption was associated with a decreased risk of urolithiasis [118,119,120], while others reported that tea consumption increased the risk of stone formation [121]. Rode et al. explored the effects of green tea consumption in hypercalciuric stone formers and found no evidence for increased stone risk factors in daily green tea drinkers. However, they did observe that COM stones were extremely lower in green tea drinkers, accompanied by a decrease in the oxalate supersaturation index [122]. Green tea from Japan and herbal tea from South Africa (both rich in polyphenols) were administered to CaOx stone formers in a pilot study [123]. Crystal morphology showed that both tea consumption favored the tendency to change from COD to COM, which is more adherent to renal epithelial cells and not protective against stone formation [123]. Tracy et al. explored the effects of pomegranate administration on risk factors for nephrolithiasis [124]. They recruited recurrent stone formers and non-stone formers who received pomegranate polyphenol extract for 3 months. Following the treatment, the researchers observed a 10% increase in the activity of paraoxonase1, an anti-atherosclerotic enzyme with antioxidant properties, in recurrent stone formers. Additionally, there was a noticeable trend towards a reduction in the supersaturation of calcium oxalate, indicating that this intervention may effectively control the risk of renal stone formation [124]. *Hibiscus sabdariffa* and its polyphenol extracts were reported to exhibit antioxidative effects. A clinical study, in which stone formers received a cup of tea made from *Hibiscus sabdariffa* two times daily for 15 days, observed a significant increase in uric acid excretion and clearance [125]. A study from India found that administration of *Dolichos bifloru*, a medicinal plant containing quercetin, to CaOx stone formers reduced the risk of recurrence and yielded a better effect than potassium citrate in these patients [126].

## 6. Current Challenges and Future Perspectives

Overall, numerous studies have demonstrated the protective role of dietary polyphenols through multiple antioxidant mechanisms. However, current studies are still limited by certain bottlenecks and challenges, and future perspectives are proposed. First, the low bioavailability of dietary polyphenols has been a major concern. Bioavailability refers to the extent to which active polyphenols enter into circulation upon ingestion [127]. Polyphenol bioavailability is affected by several factors, including their chemical structure, food matrices, and inter-individual differences [128]. Chemical properties, such as the extent of glycosylation and esterification, determine intestinal absorption of polyphenols [129]. It is less efficient and rapid to absorb polyphenols in the form of esters and glycosides compared with aglycones and glucosides [130]. The reason could be that glycosylated polyphenols are hydrophilic, so they cannot passively diffuse through the intestine until they are hydrolyzed [131]. The presence of other food components, such as lipids, proteins, and carbohydrates, also has various influences on polyphenol bioavailability. Guo et al. observed that co-ingestion of quercetin in a high-fat breakfast improved the bioavailability of this flavonoid by increasing its absorption compared to fat-free ones in overweight men [132]. This positive effect could be attributed to the fact that polyphenol compounds are incorporated into the lipid fraction to enhance their stabilization with a sort of micellar protection transported to the gut tract [133]. A clinical study conducted by Serafini et al. found a decrease in the plasma concentration of caffeic acid and ferulic acid and a decreased antioxidant capacity in vivo of subjects consuming blueberries with the presence of milk compared to those consuming without milk, as milk protein was able to interact with polyphenols via chemical bindings [134]. Schramm et al. also showed that co-ingestion of catechin and epicatechin with a carbohydrate-rich matrix enhanced their bioavailability and antioxidant capacity in volunteers than co-ingestion with matrices in which other macronutrients (such as lipids or proteins) prevailed [135]. However, some researchers also reported conflicting results, as the interactions between polyphenols and nutritional constituents are complicated [136]. It is worth mentioning that only 5–10% of polyphenols can be directly absorbed in the small intestine, while the remaining move directly to the colon, where microbiota secrete enzymes (such as glycosidases, amidases, and esterases) to degrade them for absorption [137]. Since the gut microbiota differs significantly among individuals, polyphenol bioavailability shows a high inter-individual variability. Given that low bioavailability is the biggest obstacle for polyphenols in their clinical application, several strategies have been conducted to improve bioavailability. Micro- or nano-delivery systems show great potential with the use of emulsions, liposomes, and hydrogels for the protection of polyphenols during metabolic processes [138]. Oil-in-water is the most commonly used emulsion to encapsulate polyphenols to enhance their bioavailability, such as quercetin [139]. Liposomes can serve as nanocarriers for polyphenols, as they protect polyphenols against hydrolysis during digestion processes and allow them to reach target sites [140]. Hydrogels enhance the stability and bioavailability of polyphenols due to their interior porous three-dimensional polymer networks [141]. Structural modifications of the parent compound have also been reported to improve the low bioavailability. Hydrophobic flavonoids that undergo glycosylation and glucuronide conjugation can significantly change their physicochemical properties to achieve better absorption [142]. The pharmacokinetic properties of various polyphenols have been improved by adding new polar groups or masking selective functional groups in their structural skeletons [143]. However, the effectiveness of these strategies is still under investigation, and more research is needed to make any conclusive statements. Additionally, more studies are required to elucidate the accurate pharmacokinetics and metabolic dynamics of dietary polyphenols. 

Second, we cannot ignore the interaction between dietary polyphenols and other bioactive compounds. Polyphenols in tea, such as catechin, can bind to caffeine to form complexes via hydrophobic interaction and hydrogen bonding [144]. The polyphenol–caffeine complexes were found to be associated with an increase in the concentration of catechins in solution [145]. A randomized controlled trial showed that the protective vascular effects of cocoa flavanols were increased with the co-ingestion of caffeine [146]. Nakagawa et al. found that the bioavailability of ECGC was enhanced when consumed with caffeine, as caffeine might inhibit the conjugation reactions of ECGC [147]. Some studies have demonstrated that polyphenols and carotenoids can interact synergically in certain combinations to suppress proinflammatory pathways. For example, the synergistic inhibitory effect on the secretion of inflammatory mediators was confirmed when polyphenols (curcumin or carnosic acid) were combined with some carotenoids (beta-carotene, lutein, and lycopene) [148]. Calniquer et al. also showed that polyphenols and carotenoids played synergistic roles in suppressing OS via inhibition of the NF-κB pathway and activation of the Nrf2 pathway [149]. When interpreting the beneficial effects of dietary polyphenols in many clinical studies, whether the interventions were based on consumption of whole foods, dietary supplements, purified extracts, or isolated compounds must be taken into account [150]. Indeed, the effects of the interaction between polyphenols and other bioactive compounds should be carefully evaluated. Advantageous complementary, additive, or synergistic effects, as well as negative or neutralizing effects, are situations that need attention. Thus, it is worthy to explore the potentiality of such complex interactions with properly designed in vitro, in vivo, and human intervention studies.

Third, current information about the diversity and concentration of polyphenol compounds in plant foods was inadequate. Standardized analytical methods are limited to comprehensively characterize and quantify the diverse polyphenols in plant foods [151]. The available information has been collected from heterogeneous sources, in which the original food sampling and description are uncertain, making it difficult to integrate [152]. What is more, the uneven distribution of polyphenol compounds in different parts of plants further complicates the quantification [153]. For example, quercetin is primarily found in apple peels, and it is not present in peeled fruit [154]. As there is limited information regarding their levels of particular foods, it is difficult to figure out their total intake fully, making it challenging to provide dietary recommendations for polyphenols intake. Unlike synthetic drugs, there is currently no legislation regulating polyphenol supplementation and consumption. Thus, the exact level of polyphenols in regular diets remains a subject of research. In the future, food records and labels for quantitative information about polyphenols need to be developed to allow consumers to quickly and accurately evaluate total intake and required precautionary measures.

Fourth, despite their protective effects on urolithiasis, the potential adverse effects of dietary polyphenols also need attention, especially side effects on the kidneys. In rats with adenine-induced chronic renal failure, green tea intake was shown to increase serum levels of two nephro-cardiovascular toxins, endogenous indoxyl sulfate and p-cresyl sulfate, as well as serum levels of creatinine and urea nitrogen [155]. Catechins in green tea are putative substrates of renal organic anion transporters, such as OAT1 and OAT3. The authors concluded that green tea metabolites inhibit the uptake transporting functions of OAT1 and OAT3 to reduce the renal excretion of nephro-cardiovascular toxins, further impairing renal function [155]. Similarly, Murakami et al. found that colitis mice treated with feed at 1% catechin showed a marked increase in serum creatinine level [156]. Some polyphenols may have carcinogenic effects on the kidney at high doses. For example, rats and mice exposed to 2% caffeic acid in their diets could develop kidney tumors [157]. Quercetin can suppress the O-methylation of catecholestrogens and increase kidney concentrations of 2- and 4-hydroxyestrodiol, leading to estradiol-induced tumorigenesis [158]. Studies have suggested that the safety of polyphenol consumption depends on the level and duration of intake. A high dose of certain polyphenols taken for an extended period may lead to severe toxic effects [159]. Hence, it is important to recognize that the usage of polyphenols depends on their benefits versus risks [160]. As the dose and duration of intake distinguish a poison from a remedy, human clinical trials assessing the safety of polyphenols with antiurolithiatic effects should be conducted.

Last but not least, although the molecular mechanisms of polyphenols have been widely explored in vitro and in vivo, there have been limited pre-clinical and clinical investigations. Polyphenols often possess potent antioxidant activity either in vitro or in vivo studies but fail to demonstrate efficacy in clinical trials. Differences in genetic composition between humans and animals might account for this variation [161]. Another reason could be that the doses used in in vitro studies do not completely reflect the true in vivo conditions of patients [162]. Thus, the translational applications of polyphenols as potential antiurolithiatic therapeutic agents remain to be addressed due to inadequate convincing evidence from human studies. Instead of relying on in vitro or animal findings for conclusions, clinical studies on the bioavailability, efficacy, safety, and exact mechanisms of action should be conducted to make the findings more robust. We believe that dietary polyphenol interventions will play a pivotal role in the treatment and prevention of urolithiasis in the future.

## Figures and Tables

**Table 1:** Different groups and subgroups of dietary polyphenols and their sources.

Dietary Polyphenols Groups	Dietary Polyphenols Subgroups	Example	Dietary Source
Flavonoids	Flavones	Luteolin, Apigenin, Chrysin, Vitexin	Parsley, Celery, Thyme, Capsicum pepper
	Flavonols	Quercetin, Hyperoside, Kaempferol, Myricetin, Galangin, Fisetin	Red cabbage, Onion, Leek, Curly pale, Cherry, Tomato, Broccoli, Blueberry, Apricot, Apple, Black Grape, Green and black tea, Beans, Red wine
	Flavanones	Hesperetin, Naringenin, Eriodictyol, Diosmin, Isosakuranetin	Orange, Grapefruit, Lemon juice
	Flavanols	(Epi)Catechin, (Epi)Gallocatechin, Epigallocatechin gallate, Theaflavin	Green and black tea, Cocoa, Chocolates, Apricots, Beans, Grapes, Berries, Apples, Red wine
	Isoflavones	Genistein, Genistin, Daidzein, Daidzin, Biochanin A, Puerarin, Formononetin	Soybeans, Soy foods, Legumes
	Anthocyanins	Cyanidin, Delphinidin, Pelargonidin, Peonidin, Petunidin, Malvidin	Red, blue, and purple berries, Red and purple grapes, Red wine, Cherry, Rhubarb
Phenolic acids	Hydroxybenzoic acid	Gallic acid, Protocatechuic acid	Blackberry, Raspberry, Strawberry, Black currant
	Hydroxycinnamic acid	Chlorogenic acid, Ferulic acid, Caffeic acid, Coumaric acid	Blueberry, Kiwi, Cherry, Plum, Apple, Pear, Peach, Chicory, Artichoke, Potato, Coffee
Stilbenes	-	Resveratrol	Grapes, Red wine, Pomegranate, Groundnut
Lignans	-	Secoisolariciresinol	Linseed, Lentils, Garlic, Asparagus, Carrots, Pears, Prunes

**Table 2:** Cellular and animal studies on the antioxidant effects of polyphenols compounds on urolithiasis.

Polyphenols Compounds	Experimental Model	Mechanisms of Action	References
Apigenin	Wistar rats drink water containing 0.75% ethylene glycol and 1% ammonium chloride.	Inhibition of the TGF-β pathway	[81]
Vitexin	C57BL/6 mice with intraperitoneal injection of 100 mg/kg/d glyoxylate. HK-2 cells treated with COM. THP-1 cells treated with COM.	Inhibition of pyroptosis, apoptosis, epithelial–mesenchymal transition, and macrophage infiltration	[82]
Quercetin	SD rats fed chow containing 3% sodium oxalate. MDCK cells treated with sodium oxalate.	Inhibition of lipid peroxidation, Activation of SOD and CAT activities	[83]
Quercetin	Wistar rats drink water containing 1% ethylene glycol.	Inhibition of the p38-MAPK pathway	[84]
Hyperoside	HK-2 cells treated with oxalate.	Activation of the Nrf2/HO-1/NQO1 pathway	[86]
Quercetin+Hyperoside	SD rats drink water containing 0.5% ethylene glycol.	-	[87]
Kaempferol	C57BL/6 mice with intraperitoneal injection of 100 mg/kg/d glyoxylate. HK-2 cells treated with COM.	Inhibition of the AR/NOX2 pathway	[88]
Catechin	Wistar rats drink water containing 5% ethylene glycol. NRK-52E cells treated with COM.	Inhibition of the changes in mitochondrial membrane potential, Inhibition of lipid peroxidation, Inhibition of apoptosis	[89]
Epigallocatechin gallate	SD rats fed chow containing 3% sodium oxalate. NRK-52E cells treated with oxalate.	-	[61]
Epigallocatechin gallate	MDCK cells treated with COM.	Inhibition of oxidized protein expression	[93]
Theaflavin	SD rats drink water containing 0.8% ethylene glycol and 0.8% ammonium chloride. HK-2 cells treated with COM	Regulation of the miR-128/SIRT1 axis	[94]
Puerarin	C57BL/6 mice with intraperitoneal injection of 100 mg/kg/d glyoxylate. HK-2 Cells treated with COM.	Activation of the SIRT1/AKT/p38 pathway	[95]
Gallic acid	C57BL/6 mice with intraperitoneal injection of 75 mg/kg/d glyoxylate. HK-2 cells treated with COM.	Activation of the Nrf2/HO-1 pathway	[96]
Chlorogenic acid	SD rats drink water containing 1% ethylene glycol.	Inhibition of the NF-κB/Runx2/AP-1/Osterix pathway	[97]
Resveratrol	SD rats drink water containing 0.8% ethylene glycol and 1% ammonium chloride. Human primary renal epithelial cells treated with oxalate.	Inhibition of NADPH oxidase subunits (p22phox and p47phox), MCP-1, OPN, TGF-1, TGFR-I/II and hyaluronan expression	[100]
Resveratrol	SD rats with intraperitoneal injection of 100 mg/kg/day glyoxylate. NRK-52E cells treated with oxalate.	Activation of a TFEB-induced autophagy	[102]
Curcumin	C57BL/6 mice with intraperitoneal injection of 100 mg/kg/d glyoxylate.	Activation of the Nrf2/HO-1/NQO1 pathway	[104]

## Data Availability

Not applicable.

## References

[1] Khan S.R., Pearle M.S., Robertson W.G., Gambaro G., Canales B.K., Doizi S., Traxer O., Tiselius H.G. (2016). Kidney stones. Nat. Rev. Dis. Primers.

[2] Sorokin I., Mamoulakis C., Miyazawa K., Rodgers A., Talati J., Lotan Y. (2017). Epidemiology of stone disease across the world. World J. Urol..

[3] Scales C.D., Smith A.C., Hanley J.M., Saigal C.S. (2012). Prevalence of kidney stones in the United States. Eur. Urol..

[4] D’Costa M.R., Haley W.E., Mara K.C., Enders F.T., Vrtiska T.J., Pais V.M., Jacobsen S.J., McCollough C.H., Lieske J.C., Rule A.D. (2019). Symptomatic and Radiographic Manifestations of Kidney Stone Recurrence and Their Prediction by Risk Factors: A Prospective Cohort Study. J. Am. Soc. Nephrol..

[5] Antonelli J.A., Maalouf N.M., Pearle M.S., Lotan Y. (2014). Use of the National Health and Nutrition Examination Survey to calculate the impact of obesity and diabetes on cost and prevalence of urolithiasis in 2030. Eur. Urol..

[6] Wignall G.R., Canales B.K., Denstedt J.D., Monga M. (2008). Minimally invasive approaches to upper urinary tract urolithiasis. Urol. Clin. North Am..

[7] Singh P., Enders F.T., Vaughan L.E., Bergstralh E.J., Knoedler J.J., Krambeck A.E., Lieske J.C., Rule A.D. (2015). Stone Composition Among First-Time Symptomatic Kidney Stone Formers in the Community. Mayo Clin. Proc..

[8] Finlayson B. (1978). Physicochemical aspects of urolithiasis. Kidney Int..

[9] Bushinsky D.A., Frick K.K., Nehrke K. (2006). Genetic hypercalciuric stone-forming rats. Curr. Opin. Nephrol. Hypertens.

[10] Khan S.R., Canales B.K. (2011). Ultrastructural investigation of crystal deposits in Npt2a knockout mice: Are they similar to human Randall’s plaques?. J. Urol..

[11] Mandel N. (1994). Crystal-membrane interaction in kidney stone disease. J. Am. Soc. Nephrol..

[12] Khan S.R., Byer K.J., Thamilselvan S., Hackett R.L., McCormack W.T., Benson N.A., Vaughn K.L., Erdos G.W. (1999). Crystal-cell interaction and apoptosis in oxalate-associated injury of renal epithelial cells. J. Am. Soc. Nephrol..

[13] Daudon M., Bazin D., Letavernier E. (2015). Randall’s plaque as the origin of calcium oxalate kidney stones. Urolithiasis.

[14] Khan S.R., Canales B.K., Dominguez-Gutierrez P.R. (2021). Randall’s plaque and calcium oxalate stone formation: Role for immunity and inflammation. Nat. Rev. Nephrol..

[15] Khan S.R. (2014). Reactive oxygen species, inflammation and calcium oxalate nephrolithiasis. Transl. Androl. Urol..

[16] Mithul Aravind S., Wichienchot S., Tsao R., Ramakrishnan S., Chakkaravarthi S. (2021). Role of dietary polyphenols on gut microbiota, their metabolites and health benefits. Food Res. Int..

[17] Rudrapal M., Khairnar S.J., Khan J., Dukhyil A.B., Ansari M.A., Alomary M.N., Alshabrmi F.M., Palai S., Deb P.K., Devi R. (2022). Dietary Polyphenols and Their Role in Oxidative Stress-Induced Human Diseases: Insights Into Protective Effects, Antioxidant Potentials and Mechanism(s) of Action. Front. Pharmacol..

[18] Robbins R.J. (2003). Phenolic acids in foods: An overview of analytical methodology. J. Agric. Food Chem..

[19] Wan M.L.Y., Co V.A., El-Nezami H. (2021). Dietary polyphenol impact on gut health and microbiota. Crit. Rev. Food. Sci. Nutr..

[20] Zhang H., Tsao R. (2016). Dietary polyphenols, oxidative stress and antioxidant and anti-inflammatory effects. Curr. Opin. Food Sci..

[21] Khan S.R. (2013). Reactive oxygen species as the molecular modulators of calcium oxalate kidney stone formation: Evidence from clinical and experimental investigations. J. Urol..

[22] Sies H., Belousov V.V., Chandel N.S., Davies M.J., Jones D.P., Mann G.E., Murphy M.P., Yamamoto M., Winterbourn C. (2022). Defining roles of specific reactive oxygen species (ROS) in cell biology and physiology. Nat. Rev. Mol. Cell Biol..

[23] Roy Z., Bansal R., Siddiqui L., Chaudhary N. (2023). Understanding the Role of Free Radicals and Antioxidant Enzymes in Human Diseases. Curr. Pharm. Biotechnol..

[24] Manea A. (2010). NADPH oxidase-derived reactive oxygen species: Involvement in vascular physiology and pathology. Cell Tissue Res..

[25] Ray P.D., Huang B.W., Tsuji Y. (2012). Reactive oxygen species (ROS) homeostasis and redox regulation in cellular signaling. Cell Signal.

[26] Li N., Yi F.X., Spurrier J.L., Bobrowitz C.A., Zou A.P. (2002). Production of superoxide through NADH oxidase in thick ascending limb of Henle’s loop in rat kidney. Am. J. Physiol. Renal. Physiol..

[27] Geiszt M., Kopp J.B., Várnai P., Leto T.L. (2000). Identification of renox, an NAD(P)H oxidase in kidney. Proc. Natl. Acad. Sci. USA.

[28] Leto T.L., Morand S., Hurt D., Ueyama T. (2009). Targeting and regulation of reactive oxygen species generation by Nox family NADPH oxidases. Antioxid. Redox. Signal.

[29] Sedeek M., Nasrallah R., Touyz R.M., Hébert R.L. (2013). NADPH oxidases, reactive oxygen species, and the kidney: Friend and foe. J. Am. Soc. Nephrol..

[30] Joshi S., Peck A.B., Khan S.R. (2013). NADPH oxidase as a therapeutic target for oxalate induced injury in kidneys. Oxid Med. Cell Longev..

[31] Kawahara T., Quinn M.T., Lambeth J.D. (2007). Molecular evolution of the reactive oxygen-generating NADPH oxidase (Nox/Duox) family of enzymes. BMC Evol. Biol..

[32] Andreyev A.Y., Kushnareva Y.E., Starkov A.A. (2005). Mitochondrial metabolism of reactive oxygen species. Biochemistry.

[33] D’Autréaux B., Toledano M.B. (2007). ROS as signalling molecules: Mechanisms that generate specificity in ROS homeostasis. Nat. Rev. Mol. Cell Biol..

[34] Scherz-Shouval R., Elazar Z. (2007). ROS, mitochondria and the regulation of autophagy. Trends Cell Biol..

[35] Baggio B., Gambaro G., Ossi E., Favaro S., Borsatti A. (1983). Increased urinary excretion of renal enzymes in idiopathic calcium oxalate nephrolithiasis. J. Urol..

[36] Boonla C., Wunsuwan R., Tungsanga K., Tosukhowong P. (2007). Urinary 8-hydroxydeoxyguanosine is elevated in patients with nephrolithiasis. Urol. Res..

[37] Schwille P.O., Manoharan M., Schmiedl A. (2005). Is idiopathic recurrent calcium urolithiasis in males a cellular disease? Laboratory findings in plasma, urine and erythrocytes, emphasizing the absence and presence of stones, oxidative and mineral metabolism: An observational study. Clin. Chem. Lab. Med..

[38] Holoch P.A., Tracy C.R. (2011). Antioxidants and self-reported history of kidney stones: The National Health and Nutrition Examination Survey. J. Endourol..

[39] Coe F.L., Evan A.P., Lingeman J.E., Worcester E.M. (2010). Plaque and deposits in nine human stone diseases. Urol. Res..

[40] Linnes M.P., Krambeck A.E., Cornell L., Williams J.C., Korinek M., Bergstralh E.J., Li X., Rule A.D., McCollough C.M., Vrtiska T.J. (2013). Phenotypic characterization of kidney stone formers by endoscopic and histological quantification of intrarenal calcification. Kidney Int..

[41] Khan S.R., Rodriguez D.E., Gower L.B., Monga M. (2012). Association of Randall plaque with collagen fibers and membrane vesicles. J. Urol..

[42] Khan S.R., Glenton P.A., Byer K.J. (2006). Modeling of hyperoxaluric calcium oxalate nephrolithiasis: Experimental induction of hyperoxaluria by hydroxy-L-proline. Kidney Int..

[43] Zuo J., Khan A., Glenton P.A., Khan S.R. (2011). Effect of NADPH oxidase inhibition on the expression of kidney injury molecule and calcium oxalate crystal deposition in hydroxy-L-proline-induced hyperoxaluria in the male Sprague-Dawley rats. Nephrol. Dial. Transplant.

[44] Thamilselvan S., Hackett R.L., Khan S.R. (1997). Lipid peroxidation in ethylene glycol induced hyperoxaluria and calcium oxalate nephrolithiasis. J. Urol..

[45] McKee M.D., Nanci A., Khan S.R. (1995). Ultrastructural immunodetection of osteopontin and osteocalcin as major matrix components of renal calculi. J. Bone Miner Res..

[46] de Water R., Noordermeer C., van der Kwast T.H., Nizze H., Boevé E.R., Kok D.J., Schröder F.H. (1999). Calcium oxalate nephrolithiasis: Effect of renal crystal deposition on the cellular composition of the renal interstitium. Am. J Kidney Dis..

[47] Khan S.R., Khan A., Byer K.J. (2011). Temporal changes in the expression of mRNA of NADPH oxidase subunits in renal epithelial cells exposed to oxalate or calcium oxalate crystals. Nephrol. Dial. Transplant.

[48] Thamilselvan V., Menon M., Thamilselvan S. (2012). Selective Rac1 inhibition protects renal tubular epithelial cells from oxalate-induced NADPH oxidase-mediated oxidative cell injury. Urol. Res..

[49] Thamilselvan V., Menon M., Thamilselvan S. (2009). Oxalate-induced activation of PKC-alpha and -delta regulates NADPH oxidase-mediated oxidative injury in renal tubular epithelial cells. Am. J. Physiol. Renal. Physiol..

[50] Byer K., Khan S.R. (2005). Citrate provides protection against oxalate and calcium oxalate crystal induced oxidative damage to renal epithelium. J. Urol..

[51] Lieske J.C., Hammes M.S., Hoyer J.R., Toback F.G. (1997). Renal cell osteopontin production is stimulated by calcium oxalate monohydrate crystals. Kidney Int..

[52] Umekawa T., Chegini N., Khan S.R. (2002). Oxalate ions and calcium oxalate crystals stimulate MCP-1 expression by renal epithelial cells. Kidney Int..

[53] Asselman M., Verhulst A., De Broe M.E., Verkoelen C.F. (2003). Calcium oxalate crystal adherence to hyaluronan-, osteopontin-, and CD44-expressing injured/regenerating tubular epithelial cells in rat kidneys. J. Am. Soc. Nephrol..

[54] Hong S.Y., Xia Q.D., Xu J.Z., Liu C.Q., Sun J.X., Xun Y., Wang S.G. (2022). Identification of the pivotal role of SPP1 in kidney stone disease based on multiple bioinformatics analysis. BMC Med. Genomics.

[55] Verhulst A., Asselman M., Persy V.P., Schepers M.S., Helbert M.F., Verkoelen C.F., De Broe M.E. (2003). Crystal retention capacity of cells in the human nephron: Involvement of CD44 and its ligands hyaluronic acid and osteopontin in the transition of a crystal binding- into a nonadherent epithelium. J. Am. Soc. Nephrol..

[56] Joshi S., Clapp W.L., Wang W., Khan S.R. (2015). Osteogenic changes in kidneys of hyperoxaluric rats. Biochim. Biophys Acta.

[57] Okada A., Yasui T., Hamamoto S., Hirose M., Kubota Y., Itoh Y., Tozawa K., Hayashi Y., Kohri K. (2009). Genome-wide analysis of genes related to kidney stone formation and elimination in the calcium oxalate nephrolithiasis model mouse: Detection of stone-preventive factors and involvement of macrophage activity. J. Bone Miner Res..

[58] Talham D.R., Backov R., Benitez I.O., Sharbaugh D.M., Whipps S., Khan S.R. (2006). Role of lipids in urinary stones: Studies of calcium oxalate precipitation at phospholipid langmuir monolayers. Langmuir.

[59] Huang H.S., Chen J., Chen C.F., Ma M.C. (2006). Vitamin E attenuates crystal formation in rat kidneys: Roles of renal tubular cell death and crystallization inhibitors. Kidney Int..

[60] Thamilselvan S., Menon M. (2005). Vitamin E therapy prevents hyperoxaluria-induced calcium oxalate crystal deposition in the kidney by improving renal tissue antioxidant status. BJU Int..

[61] Jeong B.C., Kim B.S., Kim J.I., Kim H.H. (2006). Effects of green tea on urinary stone formation: An in vivo and in vitro study. J. Endourol..

[62] de Paulo Farias D., de Araújo F.F., Neri-Numa I.A., Pastore G.M. (2021). Antidiabetic potential of dietary polyphenols: A mechanistic review. Food Res. Int..

[63] Khan J., Deb P.K., Priya S., Medina K.D., Devi R., Walode S.G., Rudrapal M. (2021). Dietary Flavonoids: Cardioprotective Potential with Antioxidant Effects and Their Pharmacokinetic, Toxicological and Therapeutic Concerns. Molecules.

[64] Liga S., Paul C., Péter F. (2023). Flavonoids: Overview of Biosynthesis, Biological Activity, and Current Extraction Techniques. Plants.

[65] Jucá M.M., Cysne Filho F.M.S., de Almeida J.C., Mesquita D.D.S., Barriga J.R.M., Dias K.C.F., Barbosa T.M., Vasconcelos L.C., Leal L., Ribeiro J.E. (2020). Flavonoids: Biological activities and therapeutic potential. Nat. Prod Res..

[66] Durazzo A., Lucarini M., Souto E.B., Cicala C., Caiazzo E., Izzo A.A., Novellino E., Santini A. (2019). Polyphenols: A concise overview on the chemistry, occurrence, and human health. Phytother. Res..

[67] Chong J., Poutaraud A., Hugueney P. (2009). Metabolism and roles of stilbenes in plants. Plant Sci..

[68] Wahab A., Gao K., Jia C., Zhang F., Tian G., Murtaza G., Chen J. (2017). Significance of Resveratrol in Clinical Management of Chronic Diseases. Molecules.

[69] Sok D.E., Cui H.S., Kim M.R. (2009). Isolation and bioactivities of furfuran type lignan compounds from edible plants. Recent Pat. Food Nutr. Agric..

[70] Zheng J., Cheng J., Zheng S., Feng Q., Xiao X. (2018). Curcumin, A Polyphenolic Curcuminoid With Its Protective Effects and Molecular Mechanisms in Diabetes and Diabetic Cardiomyopathy. Front. Pharmacol..

[71] Serrano J., Puupponen-Pimiä R., Dauer A., Aura A.M., Saura-Calixto F. (2009). Tannins: Current knowledge of food sources, intake, bioavailability and biological effects. Mol. Nutr. Food Res..

[72] La Rosa G., Lonardo M.S., Cacciapuoti N., Muscariello E., Guida B., Faraonio R., Santillo M., Damiano S. (2023). Dietary Polyphenols, Microbiome, and Multiple Sclerosis: From Molecular Anti-Inflammatory and Neuroprotective Mechanisms to Clinical Evidence. Int. J. Mol. Sci..

[73] Fanaro G.B., Marques M.R., Calaza K.D.C., Brito R., Pessoni A.M., Mendonça H.R., Lemos D.E.A., de Brito Alves J.L., de Souza E.L., Cavalcanti Neto M.P. (2023). New Insights on Dietary Polyphenols for the Management of Oxidative Stress and Neuroinflammation in Diabetic Retinopathy. Antioxidants.

[74] Manach C., Donovan J.L. (2004). Pharmacokinetics and metabolism of dietary flavonoids in humans. Free Radic. Res..

[75] Di Meo F., Lemaur V., Cornil J., Lazzaroni R., Duroux J.L., Olivier Y., Trouillas P. (2013). Free radical scavenging by natural polyphenols: Atom versus electron transfer. J. Phys. Chem. A.

[76] Lakey-Beitia J., Burillo A.M., La Penna G., Hegde M.L., Rao K.S. (2021). Polyphenols as Potential Metal Chelation Compounds against Alzheimer’s Disease. J. Alzheimers Dis..

[77] Akhlaghi M., Bandy B. (2009). Mechanisms of flavonoid protection against myocardial ischemia-reperfusion injury. J. Mol. Cell Cardiol..

[78] Suraweera T.L., Rupasinghe H.V., Dellaire G., Xu Z. (2020). Regulation of Nrf2/ARE Pathway by Dietary Flavonoids: A Friend or Foe for Cancer Management?. Antioxidants.

[79] Testai L. (2015). Flavonoids and mitochondrial pharmacology: A new paradigm for cardioprotection. Life Sci..

[80] Ozgová S., Hermánek J., Gut I. (2003). Different antioxidant effects of polyphenols on lipid peroxidation and hydroxyl radicals in the NADPH-, Fe-ascorbate- and Fe-microsomal systems. Biochem. Pharmacol..

[81] Azimi A., Eidi A., Mortazavi P., Rohani A.H. (2021). Protective effect of apigenin on ethylene glycol-induced urolithiasis via attenuating oxidative stress and inflammatory parameters in adult male Wistar rats. Life Sci..

[82] Ding T., Zhao T., Li Y., Liu Z., Ding J., Ji B., Wang Y., Guo Z. (2021). Vitexin exerts protective effects against calcium oxalate crystal-induced kidney pyroptosis in vivo and in vitro. Phytomedicine.

[83] Park H.K., Jeong B.C., Sung M.K., Park M.Y., Choi E.Y., Kim B.S., Kim H.H., Kim J.I. (2008). Reduction of oxidative stress in cultured renal tubular cells and preventive effects on renal stone formation by the bioflavonoid quercetin. J. Urol..

[84] Guzel A., Yunusoglu S., Calapoglu M., Candan I.A., Onaran I., Oncu M., Ergun O., Oksay T. (2021). Protective Effects of Quercetin on Oxidative Stress-Induced Tubular Epithelial Damage in the Experimental Rat Hyperoxaluria Model. Medicina.

[85] Gamero-Estevez E., Andonian S., Jean-Claude B., Gupta I., Ryan A.K. (2019). Temporal Effects of Quercetin on Tight Junction Barrier Properties and Claudin Expression and Localization in MDCK II Cells. Int. J. Mol. Sci..

[86] Chen Y., Ye L., Li W., Li D., Li F. (2018). Hyperoside protects human kidney-2 cells against oxidative damage induced by oxalic acid. Mol. Med. Rep..

[87] Zhu W., Xu Y.F., Feng Y., Peng B., Che J.P., Liu M., Zheng J.H. (2014). Prophylactic effects of quercetin and hyperoside in a calcium oxalate stone forming rat model. Urolithiasis.

[88] Yuan P., Sun X., Liu X., Hutterer G., Pummer K., Hager B., Ye Z., Chen Z. (2021). Kaempferol alleviates calcium oxalate crystal-induced renal injury and crystal deposition via regulation of the AR/NOX2 signaling pathway. Phytomedicine.

[89] Zhai W., Zheng J., Yao X., Peng B., Liu M., Huang J., Wang G., Xu Y. (2013). Catechin prevents the calcium oxalate monohydrate induced renal calcium crystallization in NRK-52E cells and the ethylene glycol induced renal stone formation in rat. BMC Complement Altern. Med..

[90] Li X., Wu G., Shang P., Bao J., Lu J., Yue Z. (2015). Anti-nephrolithic potential of catechin in melamine-related urolithiasis via the inhibition of ROS, apoptosis, phospho-p38, and osteopontin in male Sprague-Dawley rats. Free Radic Res..

[91] Grases F., Prieto R.M., Gomila I., Sanchis P., Costa-Bauzá A. (2009). Phytotherapy and renal stones: The role of antioxidants. A pilot study in Wistar rats. Urol. Res..

[92] Kanlaya R., Singhto N., Thongboonkerd V. (2016). EGCG decreases binding of calcium oxalate monohydrate crystals onto renal tubular cells via decreased surface expression of alpha-enolase. J. Biol. Inorg. Chem..

[93] Fong-Ngern K., Vinaiphat A., Thongboonkerd V. (2017). Microvillar injury in renal tubular epithelial cells induced by calcium oxalate crystal and the protective role of epigallocatechin-3-gallate. Faseb. J..

[94] Ye T., Yang X., Liu H., Lv P., Lu H., Jiang K., Peng E., Ye Z., Chen Z., Tang K. (2021). Theaflavin protects against oxalate calcium-induced kidney oxidative stress injury via upregulation of SIRT1. Int. J. Biol. Sci..

[95] Jing G.H., Liu Y.D., Liu J.N., Jin Y.S., Yu S.L., An R.H. (2022). Puerarin prevents calcium oxalate crystal-induced renal epithelial cell autophagy by activating the SIRT1-mediated signaling pathway. Urolithiasis.

[96] Zhou D., Wu Y., Yan H., Shen T., Li S., Gong J., Li G., Mai H., Wang D., Tan X. (2022). Gallic acid ameliorates calcium oxalate crystal-induced renal injury via upregulation of Nrf2/HO-1 in the mouse model of stone formation. Phytomedicine.

[97] Hoseinynejad K., Mard S.A., Mansouri Z., Lamoochi Z., Kazemzadeh R. (2022). Efficacy of chlorogenic acid against ethylene glycol-induced renal stone model: The role of NFKB-RUNX2-AP1-OSTERIX signaling pathway. Tissue Cell.

[98] Yasir F., Wahab A.T., Choudhary M.I. (2018). Protective effect of dietary polyphenol caffeic acid on ethylene glycol-induced kidney stones in rats. Urolithiasis.

[99] Nile S.H., Keum Y.S., Nile A.S., Kwon Y.D., Kim D.H. (2018). Potential cow milk xanthine oxidase inhibitory and antioxidant activity of selected phenolic acid derivatives. J. Biochem. Mol. Toxicol..

[100] Hong S.H., Lee H.J., Sohn E.J., Ko H.S., Shim B.S., Ahn K.S., Kim S.H. (2013). Anti-nephrolithic potential of resveratrol via inhibition of ROS, MCP-1, hyaluronan and osteopontin in vitro and in vivo. Pharmacol. Rep..

[101] Oksay T., Yunusoğlu S., Calapoğlu M., Aydın Candan I., Onaran İ., Ergün O., Özorak A. (2017). Protective impact of resveratrol in experimental rat model of hyperoxaluria. Int. Urol. Nephrol..

[102] Wu Y., Xun Y., Zhang J., Hu H., Qin B., Wang T., Wang S., Li C., Lu Y. (2021). Resveratrol Attenuates Oxalate-Induced Renal Oxidative Injury and Calcium Oxalate Crystal Deposition by Regulating TFEB-Induced Autophagy Pathway. Front Cell Dev. Biol..

[103] Ghodasara J., Pawar A., Deshmukh C., Kuchekar B. (2010). Inhibitory effect of rutin and curcumin on experimentally-induced calcium oxalate urolithiasis in rats. Pharmacognosy Res..

[104] Li Y., Zhang J., Liu H., Yuan J., Yin Y., Wang T., Cheng B., Sun S., Guo Z. (2019). Curcumin ameliorates glyoxylate-induced calcium oxalate deposition and renal injuries in mice. Phytomedicine.

[105] Lee H.J., Jeong S.J., Park M.N., Linnes M., Han H.J., Kim J.H., Lieske J.C., Kim S.H. (2012). Gallotannin suppresses calcium oxalate crystal binding and oxalate-induced oxidative stress in renal epithelial cells. Biol. Pharm Bull..

[106] Suganuma M., Saha A., Fujiki H. (2011). New cancer treatment strategy using combination of green tea catechins and anticancer drugs. Cancer Sci..

[107] Maeda K., Kuzuya M., Cheng X.W., Asai T., Kanda S., Tamaya-Mori N., Sasaki T., Shibata T., Iguchi A. (2003). Green tea catechins inhibit the cultured smooth muscle cell invasion through the basement barrier. Atherosclerosis.

[108] Itoh Y., Yasui T., Okada A., Tozawa K., Hayashi Y., Kohri K. (2005). Preventive effects of green tea on renal stone formation and the role of oxidative stress in nephrolithiasis. J Urol.

[109] Li Z., Chang L., Ren X., Hu Y., Chen Z. (2021). Modulation of Rat Kidney Stone Crystallization and the Relative Oxidative Stress Pathway by Green Tea Polyphenol. ACS Omega.

[110] Ghalayini I.F., Al-Ghazo M.A., Harfeil M.N. (2011). Prophylaxis and therapeutic effects of raspberry (Rubus idaeus) on renal stone formation in Balb/c mice. Int. Braz J. Urol..

[111] Tugcu V., Kemahli E., Ozbek E., Arinci Y.V., Uhri M., Erturkuner P., Metin G., Seckin I., Karaca C., Ipekoglu N. (2008). Protective effect of a potent antioxidant, pomegranate juice, in the kidney of rats with nephrolithiasis induced by ethylene glycol. J. Endourol..

[112] Ilbey Y.O., Ozbek E., Simsek A., Cekmen M., Somay A., Tasci A.I. (2009). Effects of pomegranate juice on hyperoxaluria-induced oxidative stress in the rat kidneys. Ren. Fail.

[113] Zhang J., Hou A., Dong J., Zheng S., Yu H., Wang X., Jiang H., Yang L. (2022). Screening out key compounds of Glechomae Herba for antiurolithic activity and quality control based on spectrum-effect relationships coupled with UPLC-QDA. Biomed. Pharmacother..

[114] Li J., Wen Q., Feng Y., Zhang J., Luo Y., Tan T. (2019). Characterization of the multiple chemical components of Glechomae Herba using ultra high performance liquid chromatography coupled to quadrupole-time-of-flight tandem mass spectrometry with diagnostic ion filtering strategy. J. Sep. Sci..

[115] Ilhan M., Ergene B., Süntar I., Ozbilgin S., Saltan Çitoğlu G., Demirel M.A., Keleş H., Altun L., Küpeli Akkol E. (2014). Preclinical Evaluation of Antiurolithiatic Activity of Viburnum opulus L. on Sodium Oxalate-Induced Urolithiasis Rat Model. Evid. Based Complement Alternat Med..

[116] Chao Y., Gao S., Li N., Zhao H., Qian Y., Zha H., Chen W., Dong X. (2020). Lipidomics Reveals the Therapeutic Effects of EtOAc Extract of Orthosiphon stamineus Benth. on Nephrolithiasis. Front. Pharmacol..

[117] Zhou J., Jin J., Li X., Zhao Z., Zhang L., Wang Q., Li J., Zhang Q., Xiang S. (2018). Total flavonoids of Desmodium styracifolium attenuates the formation of hydroxy-L-proline-induced calcium oxalate urolithiasis in rats. Urolithiasis.

[118] Chen H.Y., Wu J.S., Chang Y.F., Sun Z.J., Chang C.J., Lu F.H., Yang Y.C. (2019). Increased amount and duration of tea consumption may be associated with decreased risk of renal stone disease. World J. Urol..

[119] Shu X., Cai H., Xiang Y.B., Li H., Lipworth L., Miller N.L., Zheng W., Shu X.O., Hsi R.S. (2019). Green tea intake and risk of incident kidney stones: Prospective cohort studies in middle-aged and elderly Chinese individuals. Int. J. Urol..

[120] Zhuo D., Li M., Cheng L., Zhang J., Huang H., Yao Y. (2019). A Study of Diet and Lifestyle and the Risk of Urolithiasis in 1519 Patients in Southern China. Med. Sci. Monit..

[121] Wu Z.B., Jiang T., Lin G.B., Wang Y.X., Zhou Y., Chen Z.Q., Xu Y.M., Ye H.B., Chen B.J., Bao X.Z. (2017). Tea Consumption is Associated with Increased Risk of Kidney Stones in Northern Chinese: A Cross-sectional Study. Biomed Environ. Sci..

[122] Rode J., Bazin D., Dessombz A., Benzerara Y., Letavernier E., Tabibzadeh N., Hoznek A., Tligui M., Traxer O., Daudon M. (2019). Daily Green Tea Infusions in Hypercalciuric Renal Stone Patients: No Evidence for Increased Stone Risk Factors or Oxalate-Dependent Stones. Nutrients.

[123] Rodgers A., Mokoena M., Durbach I., Lazarus J., de Jager S., Ackermann H., Breytenbach I., Okada A., Usami M., Hirose Y. (2016). Do teas rich in antioxidants reduce the physicochemical and peroxidative risk factors for calcium oxalate nephrolithiasis in humans? Pilot studies with Rooibos herbal tea and Japanese green tea. Urolithiasis.

[124] Tracy C.R., Henning J.R., Newton M.R., Aviram M., Bridget Zimmerman M. (2014). Oxidative stress and nephrolithiasis: A comparative pilot study evaluating the effect of pomegranate extract on stone risk factors and elevated oxidative stress levels of recurrent stone formers and controls. Urolithiasis.

[125] Prasongwatana V., Woottisin S., Sriboonlue P., Kukongviriyapan V. (2008). Uricosuric effect of Roselle (Hibiscus sabdariffa) in normal and renal-stone former subjects. J. Ethnopharmacol..

[126] Singh R.G., Behura S.K., Kumar R. (2010). Litholytic property of Kulattha (Dolichous biflorus) vs potassium citrate in renal calculus disease: A comparative study. J. Assoc. Physicians India.

[127] Lippolis T., Cofano M., Caponio G.R., De Nunzio V., Notarnicola M. (2023). Bioaccessibility and Bioavailability of Diet Polyphenols and Their Modulation of Gut Microbiota. Int. J. Mol. Sci..

[128] Russo M., Moccia S., Spagnuolo C., Tedesco I., Russo G.L. (2020). Roles of flavonoids against coronavirus infection. Chem. Biol. Interact.

[129] Scalbert A., Morand C., Manach C., Rémésy C. (2002). Absorption and metabolism of polyphenols in the gut and impact on health. Biomed Pharmacother..

[130] Eleazu C., Eleazu K., Kalu W. (2017). Management of Benign Prostatic Hyperplasia: Could Dietary Polyphenols Be an Alternative to Existing Therapies?. Front. Pharmacol..

[131] Németh K., Plumb G.W., Berrin J.G., Juge N., Jacob R., Naim H.Y., Williamson G., Swallow D.M., Kroon P.A. (2003). Deglycosylation by small intestinal epithelial cell beta-glucosidases is a critical step in the absorption and metabolism of dietary flavonoid glycosides in humans. Eur. J. Nutr..

[132] Guo Y., Mah E., Davis C.G., Jalili T., Ferruzzi M.G., Chun O.K., Bruno R.S. (2013). Dietary fat increases quercetin bioavailability in overweight adults. Mol. Nutr. Food Res..

[133] Jakobek L. (2015). Interactions of polyphenols with carbohydrates, lipids and proteins. Food Chem..

[134] Serafini M., Testa M.F., Villaño D., Pecorari M., van Wieren K., Azzini E., Brambilla A., Maiani G. (2009). Antioxidant activity of blueberry fruit is impaired by association with milk. Free Radic. Biol. Med..

[135] Schramm D.D., Karim M., Schrader H.R., Holt R.R., Kirkpatrick N.J., Polagruto J.A., Ensunsa J.L., Schmitz H.H., Keen C.L. (2003). Food effects on the absorption and pharmacokinetics of cocoa flavanols. Life Sci..

[136] Cianciosi D., Forbes-Hernández T.Y., Regolo L., Alvarez-Suarez J.M., Navarro-Hortal M.D., Xiao J., Quiles J.L., Battino M., Giampieri F. (2022). The reciprocal interaction between polyphenols and other dietary compounds: Impact on bioavailability, antioxidant capacity and other physico-chemical and nutritional parameters. Food Chem..

[137] Carrera-Quintanar L., López Roa R.I., Quintero-Fabián S., Sánchez-Sánchez M.A., Vizmanos B., Ortuño-Sahagún D. (2018). Phytochemicals That Influence Gut Microbiota as Prophylactics and for the Treatment of Obesity and Inflammatory Diseases. Mediators Inflamm..

[138] Aloo S.O., Ofosu F.K., Kim N.H., Kilonzi S.M., Oh D.H. (2023). Insights on Dietary Polyphenols as Agents against Metabolic Disorders: Obesity as a Target Disease. Antioxidants.

[139] Aghababaei F., Hadidi M. (2023). Recent Advances in Potential Health Benefits of Quercetin. Pharmaceuticals.

[140] Mignet N., Seguin J., Chabot G.G. (2013). Bioavailability of polyphenol liposomes: A challenge ahead. Pharmaceutics.

[141] Ning P., Lü S., Bai X., Wu X., Gao C., Wen N., Liu M. (2018). High encapsulation and localized delivery of curcumin from an injectable hydrogel. Mater. Sci. Eng. C Mater. Biol. Appl..

[142] Teng H., Chen L. (2019). Polyphenols and bioavailability: An update. Crit. Rev. Food Sci. Nutr..

[143] Zhao J., Yang J., Xie Y. (2019). Improvement strategies for the oral bioavailability of poorly water-soluble flavonoids: An overview. Int. J. Pharm..

[144] Bøhn S.K., Ward N.C., Hodgson J.M., Croft K.D. (2012). Effects of tea and coffee on cardiovascular disease risk. Food Funct..

[145] Babu V.R., Thakur M.S., Patra S. (2012). Effect of physicochemical parameters on enzymatic biodecaffeination during tea fermentation. Appl. Biochem. Biotechnol..

[146] Sansone R., Ottaviani J.I., Rodriguez-Mateos A., Heinen Y., Noske D., Spencer J.P., Crozier A., Merx M.W., Kelm M., Schroeter H. (2017). Methylxanthines enhance the effects of cocoa flavanols on cardiovascular function: Randomized, double-masked controlled studies. Am. J. Clin. Nutr..

[147] Nakagawa K., Nakayama K., Nakamura M., Sookwong P., Tsuduki T., Niino H., Kimura F., Miyazawa T. (2009). Effects of co-administration of tea epigallocatechin-3-gallate (EGCG) and caffeine on absorption and metabolism of EGCG in humans. Biosci. Biotechnol. Biochem..

[148] Bungau S., Abdel-Daim M.M., Tit D.M., Ghanem E., Sato S., Maruyama-Inoue M., Yamane S., Kadonosono K. (2019). Health Benefits of Polyphenols and Carotenoids in Age-Related Eye Diseases. Oxid. Med. Cell Longev..

[149] Calniquer G., Khanin M., Ovadia H., Linnewiel-Hermoni K., Stepensky D., Trachtenberg A., Sedlov T., Braverman O., Levy J., Sharoni Y. (2021). Combined Effects of Carotenoids and Polyphenols in Balancing the Response of Skin Cells to UV Irradiation. Molecules.

[150] Nurk E., Refsum H., Drevon C.A., Tell G.S., Nygaard H.A., Engedal K., Smith A.D. (2009). Intake of flavonoid-rich wine, tea, and chocolate by elderly men and women is associated with better cognitive test performance. J. Nutr..

[151] Ma S., Kim C., Neilson A.P., Griffin L.E., Peck G.M., O’Keefe S.F., Stewart A.C. (2019). Comparison of Common Analytical Methods for the Quantification of Total Polyphenols and Flavanols in Fruit Juices and Ciders. J. Food Sci..

[152] Spencer J.P., Abd El Mohsen M.M., Minihane A.M., Mathers J.C. (2008). Biomarkers of the intake of dietary polyphenols: Strengths, limitations and application in nutrition research. Br. J. Nutr..

[153] Shi L., Zhao W., Yang Z., Subbiah V., Suleria H.A.R. (2022). Extraction and characterization of phenolic compounds and their potential antioxidant activities. Environ. Sci. Pollut. Res. Int..

[154] Burda S., Oleszek W., Lee C.Y. (1990). Phenolic compounds and their changes in apples during maturation and cold storage. J. Agric. Food Chem..

[155] Peng Y.H., Sweet D.H., Lin S.P., Yu C.P., Lee Chao P.D., Hou Y.C. (2015). Green tea inhibited the elimination of nephro-cardiovascular toxins and deteriorated the renal function in rats with renal failure. Sci. Rep..

[156] Murakami A. (2014). Dose-dependent functionality and toxicity of green tea polyphenols in experimental rodents. Arch. Biochem. Biophys..

[157] Hagiwara A., Hirose M., Takahashi S., Ogawa K., Shirai T., Ito N. (1991). Forestomach and kidney carcinogenicity of caffeic acid in F344 rats and C57BL/6N x C3H/HeN F1 mice. Cancer Res..

[158] Zhu B.T., Liehr J.G. (1996). Inhibition of catechol O-methyltransferase-catalyzed O-methylation of 2- and 4-hydroxyestradiol by quercetin. Possible role in estradiol-induced tumorigenesis. J. Biol. Chem..

[159] Yamakoshi J., Saito M., Kataoka S., Kikuchi M. (2002). Safety evaluation of proanthocyanidin-rich extract from grape seeds. Food Chem. Toxicol..

[160] Lambert J.D., Sang S., Yang C.S. (2007). Possible controversy over dietary polyphenols: Benefits vs risks. Chem. Res. Toxicol..

[161] Boocock D.J., Faust G.E., Patel K.R., Schinas A.M., Brown V.A., Ducharme M.P., Booth T.D., Crowell J.A., Perloff M., Gescher A.J. (2007). Phase I dose escalation pharmacokinetic study in healthy volunteers of resveratrol, a potential cancer chemopreventive agent. Cancer Epidemiol. Biomarkers Prev..

[162] D’Archivio M., Filesi C., Varì R., Scazzocchio B., Masella R. (2010). Bioavailability of the polyphenols: Status and controversies. Int. J. Mol. Sci..

